# Size quantization of Dirac fermions in graphene constrictions

**DOI:** 10.1038/ncomms11528

**Published:** 2016-05-20

**Authors:** B. Terrés, L. A. Chizhova, F. Libisch, J. Peiro, D. Jörger, S. Engels, A. Girschik, K. Watanabe, T. Taniguchi, S. V. Rotkin, J. Burgdörfer, C. Stampfer

**Affiliations:** 1 JARA-FIT and 2nd Institute of Physics, RWTH Aachen University, 52056 Aachen, Germany; 2 Peter Grünberg Institute (PGI-9), Forschungszentrum Jülich, 52425 Jülich, Germany; 3 Institute for Theoretical Physics, Vienna University of Technology, 1040 Vienna, Austria; 4 National Institute for Materials Science, 1-1 Namiki, Tsukuba 305-0044, Japan; 5 Department of Physics, Lehigh University, Bethlehem, Pennsylvania 18015, USA; 6 Center for Advanced Materials and Nanotechnology, Lehigh University, Bethlehem, Pennsylvania 18015, USA; 7 Institute of Nuclear Research of the Hungarian Academy of Sciences (ATOMKI), 4001 Debrecen, Hungary

## Abstract

Quantum point contacts are cornerstones of mesoscopic physics and central building blocks for quantum electronics. Although the Fermi wavelength in high-quality bulk graphene can be tuned up to hundreds of nanometres, the observation of quantum confinement of Dirac electrons in nanostructured graphene has proven surprisingly challenging. Here we show ballistic transport and quantized conductance of size-confined Dirac fermions in lithographically defined graphene constrictions. At high carrier densities, the observed conductance agrees excellently with the Landauer theory of ballistic transport without any adjustable parameter. Experimental data and simulations for the evolution of the conductance with magnetic field unambiguously confirm the identification of size quantization in the constriction. Close to the charge neutrality point, bias voltage spectroscopy reveals a renormalized Fermi velocity of ∼1.5 × 10^6^ m s^−1^ in our constrictions. Moreover, at low carrier density transport measurements allow probing the density of localized states at edges, thus offering a unique handle on edge physics in graphene devices.

## Abstract (web summary)

Quantum point contacts are narrow electrical connections of a width comparable to the wavelength of the conducting electrons. Here, the authors create such contacts in graphene by etching constrictions and use them to identify ballistic transport and quantized conductance of size-confined Dirac fermions.

## Graphical abstract

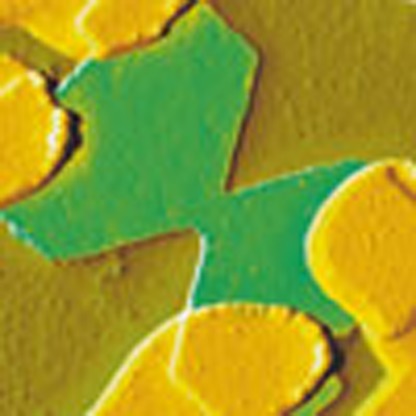

The observation of unique transport phenomena in graphene, such as Klein tunnelling^1^, evanescent wave transport^2^, or the half-integer^3^,^4^ and fractional^5^,^6^ quantum Hall effect are directly related to the material quality, as well as the relativistic dispersion of the charge carriers. As the quality of bulk graphene has been impressively improved in the last years^7^,^8^, the understanding of the role and limitations of edges on transport properties of graphene is becoming increasingly important. This is particularly true for nanoscale graphene systems where edges can dominate device properties. Indeed, the rough edges of graphene nanodevices are most probably responsible for the difficulties in observing clear confinement-induced quantization effects such as quantized conductance^9^ and shell filling^10^. So far signatures of quantized conductance have only been observed in suspended graphene, however with limited control and information on geometry and constriction width^11^. More generally, with further progress in fabrication technology, graphene nanoribbons and constrictions are expected to evolve from a disorder-dominated^12^,^13^,^14^,^15^ transport behaviour to a quasi-ballistic regime where boundary effects, crystal alignment and edge defects^16^,^17^ govern the transport characteristics. This will open the door to investigate interesting phenomena arising from edge states, including magnetic order at zig-zag edges^18^, an unusual Josephson effect^19^, unconventional edge states^20^, magnetic edge-state excitons^21^ or topologically protected quantum spin Hall states^22^.

In this work we report on the observation of size quantization and localized trap states in ballistic transport through graphene constrictions approximating quantum point contacts. Away from the Dirac point, the current features evenly spaced, reproducible kinks superposed on a linear background, in agreement with transport simulations. Scattering at the rough constriction edges reduces quantization steps to kinks in both experiment and theory. The kink spacing, and their evolution with magnetic field, allows us to unambiguously identify them as signatures of size quantization. Close to the Dirac point, deviations from ballistic behaviour allow for probing the density of localized trap states.

## Results

### Ballistic transport

**Figure 1:**
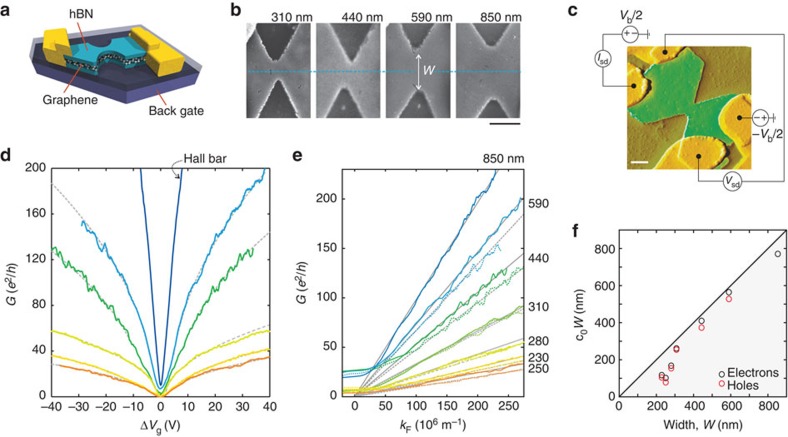
Width-dependent ballistic transport in etched graphene nanoconstrictions encapsulated in hBN. (**a**) Schematic illustration of a hBN–graphene sandwich device with the bottom and top layers of hBN appearing in green, the gold contacts in yellow, the SiO_2_ in dark blue and the Si back gate in purple. (**b**) SEM images of four investigated graphene constrictions patterned using reactive ion etching. Black scale bar, 500 nm. (**c**) False coloured atomic force microscope (AFM) image of a fabricated device. Transport is measured in a four-probe configuration to eliminate any unwanted resistance of the one-dimensional contacts^8^. The yellow colour denotes the gold contacts, green the top layer of hBN and brown the SiO_2_ substrate. White scale bar, 500 nm. (**d**) Low-bias back-gate characteristics of a Hall bar device (see arrow) and of five constriction devices with different widths ranging from 850 to 230 nm (see **e** for colour code). The dashed grey lines are fits to the data. (**e**) Low-bias four-terminal conductance of graphene quantum point contacts as function of *k*_F_ extracted in the high carrier density limit for seven different samples. The colour encodes the different samples with different constriction widths (see labels). Grey lines represent a linear fit at high values of *k*_F_, inserted as guide to the eye. Conductance deviates from the expected linear slope for small *k*_F_. Electron (hole) conductance is plotted as solid (dashed) line. Data are taken at temperatures below 2 K. (**f**) Comparison of *c*_0_*W* from conductance traces (**e**) with the width *W* (extracted from SEM images).

We prepared four-probe devices based on high-mobility graphene–hexagonal boron nitride (hBN) sandwiches on SiO_2_/Si substrates and use reactive ion etching to pattern narrow constrictions (see Methods) with widths ranging from *W*≈230 to 850 nm, connecting wide leads (Fig. 1a–c). The graphene leads are side-contacted^8^ by 80-nm-thick chrome/gold electrodes. A back-gate voltage is applied on the highly doped Si substrate to tune the carrier density in the graphene layer, 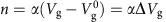, where *α* is the so-called lever arm and 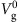 is the gate voltage of the minimum conductance, that is, the charge neutrality point. To demonstrate the high electronic quality of our graphene–hBN sandwich structures we show the gate characteristic of a reference Hall bar device (Fig. 1d and Supplementary Fig. 1). From this data we extract a carrier mobility in the range of around 150.000 cm^2^ V^−1^ s^−1^ (Supplementary Note 1), resulting in a mean free path exceeding 1 μm at around Δ*V*_g_=4.6 V. Thus, the mean free path is expected to clearly exceed all relevant length scales in our constriction devices giving rise to ballistic transport.

We measure the conductance as function of gate voltage for a number of constrictions with different widths *W* (Fig. 1d; see labels in Fig. 1e). The observed square root dependence 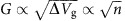 (see dashed lines in Fig. 1d) is a first indication of highly ballistic transport in our devices. Indeed, according to the Landauer theory for ballistic transport, the conductance through a perfect constriction increases by an additional conductance quantum *e*^2^/*h* whenever *Wk*_F_ reaches a multiple of *π*


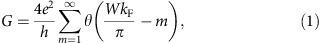


where 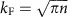 is the Fermi wave number, the factor four accounts for the valley and spin degeneracies, *θ* is the step function and we have neglected minor phase contributions due to details of the graphene edge^23^ for simplicity. Fourier expansion of equation (1) yields


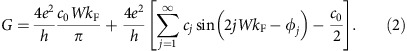


For an ideal constriction *c*_0_=1, *φ*_*j*_=0 and *c*_*j*_=1/(*jπ*), *j*>0. In the presence of edge roughness, *c*_0_ is reduced to a value below 1 due to limited average transmission, and the higher Fourier components are expected to decay in magnitude and acquire random scattering phases *φ*_*j*_≠0. Consequently, the sharp quantization steps turn into periodic modulations as will be shown below. Averaged over these modulations only the zeroth-order term in the expansion (equation (2)) survives. This mean conductance *G*^(0)^ of a constriction of width *W* thus features a linear dependenc on *k*_F_, or, equivalently, a square-root dependence as a function of back-gate voltage assuming an energy-independent transmission *c*_0_ of all modes, in accord with Fig. 1d.

By measuring the carrier-density-dependent quantum Hall effect at high magnetic fields^4^,^24^, we can independently determine the gate coupling *α* for each device (Supplementary Fig. 2, Supplementary Table 1 and Supplementary Note 2). We can thus unfold the dependence on *V*_g_ and study both the electron and hole conductance as function of *k*_F_ (Fig. 1e). From the linear slopes of *G*(*k*_F_), the product *c*_0_*W* can be extracted for each device and compared with its width *W* (Fig. 1f) determined from scanning electron microscopy (SEM) images (see, for example, Fig. 1b). The estimates for *c*_0_*W* extracted from *G*^(0)^ lie only slightly below the width *W*, where *c*_0_ decreases for decreasing width. This suggests that for the narrower devices reflections, most likely due to device geometry and edge roughness, are playing a more important role. From the data in Fig. 1f we can extract *c*_0_≈0.56 for our smallest constriction. Below we will show that, indeed, reflections at the rough edges of the constriction and not a reduction in active channel width is responsible for the deviation of the experimentally extracted *c*_0_*W* from the SEM width *W*.

### Localized states

**Figure 2:**
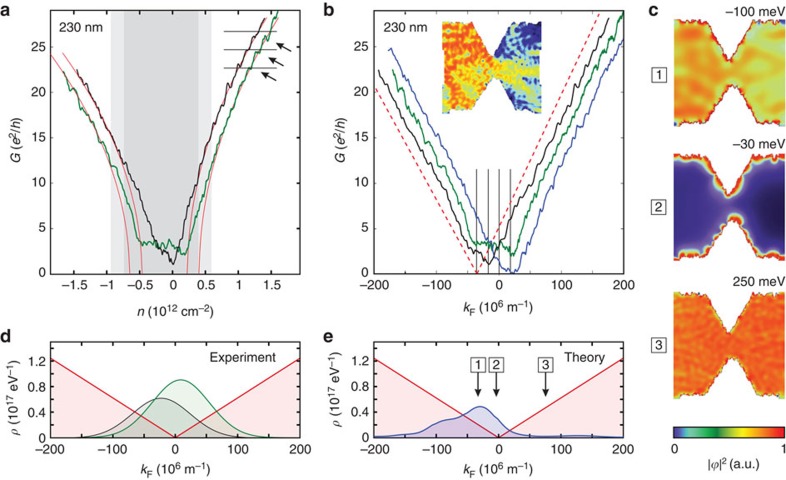
Conductance through graphene quantum point contacts. (**a**) Conductance traces of two different cool-downs (black and green curve) of the same constriction (*W*≈230 nm) as a function of charge carrier density. For the black (green) cool-down, shaded grey (light grey) regions denote deviations from the ideal Landauer model 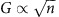 shown in red. At higher conductance values we observe well-reproduced ‘kinks' with spacings on the order of 2*e*^2^/*h* (see arrows and horizontal lines). (**b**) Experimental conductance trace as a function of *k*_F_ after correction for the density of trap states (black and green curves) and theoretical simulations of the graphene quantum point contact (blue curve). Theoretical results are rescaled to experimental device size as determined from **a**. Ideal transmission ∝*k*_F_ is shown in red as guide to the eye. Curves are offset horizontally for clarity. The inset gives an example for the probability distribution of a simulated scattering state. (**c**) Local density of states of the graphene quantum point contact from tight-binding simulations, at three different energies (−100, −30 and 250 meV; see also arrows in **e**). (**d**) Graphene density of states extracted from experiment (fit to a Gaussian) and **e** from simulation. Both experiment and theory find a substantial contribution from trap states around the Dirac point.

For small *k*_F_<50 × 10^6^ m^−1^ (that is, low carrier concentrations) the measured conductances systematically deviate from the expected linear behaviour (Fig. 1e). This deviation from the square-root relation between *G* and *n* (that is, Δ*V*_g_) becomes more apparent when focusing on *G* around the charge neutrality point (CNP). The conductance as function of *n* for two different cool-downs of the same graphene constriction (*W*≈230 nm, Fig. 2a), shows marked cool-down-dependent low-carrier-density regions with substantial deviations from 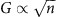. Far away from the CNP, the conductance as function of *n* for both cool-downs shows (i) an identical 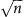 behaviour leading to the very same *c*_0_*W* and (ii) almost identical, regularly spaced kink structures (see arrows in Fig. 2a), which are, however, slightly shifted relative to another on the carrier density axis *n* (Supplementary Fig. 8). These observations suggest that the square-root relation between the Fermi wave vector *k*_F_ and the gate voltage *V*_g_, that is, *n* needs to be modified. While the quantum capacitance of ideal graphene can be neglected^25^,^26^,^27^, a small additional contribution *n*_T_(Δ*V*_g_) from, for example, localized trap states modifies the relation between *n* and *k*_F_ to


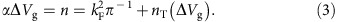


Far away from the Dirac point (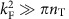), we recover the expected square-root relation. Close to the Dirac point, however, *α*Δ*V*_g_ will be strongly modified by deviations *n*_T_ from the linear density of states of ideal Dirac fermions and approaches *n*_T_(Δ*V*_g_) near the CNP. The trap states do not contribute to transport, yet they contribute to the charging characteristics^28^. Such trap states can for instance be found at the rough edges of patterned graphene devices, which feature a significant number of localized states. A tight-binding simulation of the local density of states of the experimental geometry yields a strong clustering of localized states at the device edges (Fig. 2c), which energetically lie close to the CNP (Fig. 2e). The deviation of *G* from the 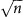 scaling also opens up the opportunity to extract *n*_T_ from experimental conductance data (for example, Fig. 2d), and thus a new pathway for device characterization. Inspired by the tight-binding simulation, we approximate the distribution of trap states as function of Fermi wave vector by a Gaussian distribution. We fit the position, height and width of the Gaussian by minimizing the difference between the measured *G*(*k*_F_) and the corresponding linear extrapolation to very low values of *k*_F_ (Fig. 2b, Supplementary Fig. 3 and Supplementary Note 3). We find good qualitative agreement between simulation and experiment (compare Fig. 2d,e). Quantitative correspondence would require a detailed, microscopic model for the trap state density *n*_T_. Note that the only difference between different traces in Fig. 2a,b,d is the exposition of the device to air for several days leading to a wider carrier density region of substantial deviations (green trace). The number of trap states (that is, the deviations around the CNP) is significantly enhanced (compare also green and black trace in Fig. 2d). As the active graphene layer is completely sandwiched in hBN, only the graphene edges are exposed to air and, very likely, experience chemical modifications. In line with our numerical results, we thus conjecture that localized states at the edges substantially contribute to *n*_T_, leading to the strong cool-down dependence we observe in our measurements. While this interpretation seems plausible and is consistent with our data, alternative explanations such as electron–hole puddles^29^ or charged impurities^13^ cannot be ruled out.

Away from the CNP our data agrees remarkably well with ballistic transport simulations through the device geometry using a modular Green's function approach^30^ (see blue trace in Fig. 2b): we simulate the four-probe constriction geometry taken from a SEM image, scaled down by a factor of four to obtain a numerically feasible problem size^31^. To account for the etched edges in the devices, we include an edge roughness amplitude of Δ*W*=0.2*W* for the constriction. This comparatively large edge roughness (which is consistent with the systematic reduction of transmission through the constriction when using the average conductance) is probably due to microcracks at the edges of the device.

### Quantized conductance

**Figure 3:**
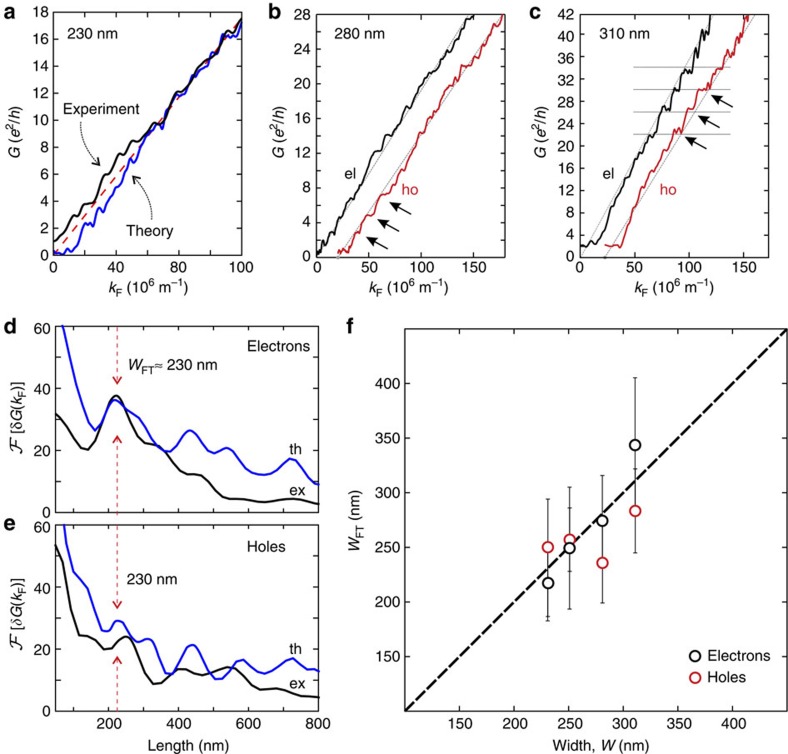
Size quantization signatures. (**a**) Comparison of the low-energy conductance between theory (blue) and experiment (black). (**b**,**c**) Measured electron (el; black trace) and hole (ho; red trace) conductance including kink or step-like structure (see arrows) as a function of *k*_F_ for two different constriction widths (see insets). The hole conductance traces are horizontally offset for clarity. (**d**) Fourier transform of the *G*−*G*^(0)^ electron conductance 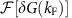 through the 230-nm graphene constriction, for experiment (ex; black trace) and theory (th; blue trace). The first peak of the Fourier transform clearly corresponds to the width *W* of the quantum point contact (marked by arrows). (**e**) Same as **d** for the hole conductance. The size of the first peak is substantially reduced for both experiment and theory due to the presence of localized states that lead to additional scattering. (**f**) Comparison of width *W*_FT_ extracted from the Fourier transform of the conductance traces (as shown in **d**,**e**) to geometric constriction width *W* from four different devices (extracted from SEM images).

Superimposed on the overall linear behaviour of *G*(*k*_F_), we find reproducible modulations (kinks) in the conductance (Fig. 3a–c and Supplementary Fig. 4). The kinks are well reproduced for several cool-downs (see arrows in Fig. 2a, Supplementary Figs 5 and 6 and Supplementary Note 4), as well as for different devices (Supplementary Fig. 7), generally showing a spacing Δ*G* varying in the range of (2−4)*e*^2^/*h* (see arrows in Fig. 3b,c). The ‘step height' and its sharpness depend on the carrier density (that is, *k*_F_), as well as on the constriction width and is strongly influenced by the overall transmission *c*_0_ (Fig. 1f). Remarkably, we observe a spacing Δ*G* of the steps close to 4*e*^2^/*h* for one of our wide samples (*W*≈310 nm) at elevated conductance values on both the electron and hole sides (see arrows and horizontal lines in Fig. 3c and Supplementary Fig. 4b)

Our assignment of the conductance ‘kinks' as signatures of quantized flow through the constriction is supported by our theoretical results. Theory and experimental data from the smallest constriction show similar smoothed, irregular modulations (Fig. 3a), instead of sharp size quantization steps^32^. The replacement of sharp quantization steps by kinks reflects the strong scattering at the rough edges of the device^33^,^34^, resulting in the accumulation of random phases in the Fourier components of *G* (equation (2)). We note that calculations with smaller edge disorder show a larger average conductance, yet very similar ‘kink' structures. As the present calculation includes only edge-disorder-induced scattering while neglecting other scattering channels such as electron–electron or electron–phonon scattering, the good agreement with the data suggests edge scattering to be the dominant contribution to the formation of the ‘kinks'. By contrast, both experimental and theoretical investigations of, for example, semiconducting GaAs heterostructures show very clear, pronounced quantization plateaus^35^. In these heterostructures, the electron wavelength near the Γ point is very long, and cannot resolve edge disorder on the nanometre scale. By contrast, K−K′ scattering in graphene allows conduction electrons to probe disorder on a much shorter length scale. Consequently, edge roughness substantially impacts transport. The comparison between experimental and theoretical data (Fig. 3a) unambiguously establishes the observed modulations to be consistent with the smoothed size quantization effects predicted by theory.

By subtracting the zeroth-order Fourier component∝*k*_F_ (or 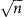), the superimposed modulations of the conductance *δG*(*k*_F_)=*G*−*G*^(0)^ provide direct information on the quantized conductance through the constriction (equation (2)). One key observation is that the Fourier transform of *δG*(*k*_F_) offers an alternative route towards the determination of the constriction width complementary to that from the mean conductance *G*^(0)^. For example, the pronounced peak of the first harmonic at 230 nm (red arrows in Fig. 3d,e) is consistent with the constriction width *W* derived from the SEM image. Our simulation also correctly reproduces the experimental observation that the peak in the Fourier spectrum of *δG*(*k*_F_) is more pronounced on the electron side (Fig. 3d) than on the hole side. This results from the slightly asymmetric energy distribution of the trap states relative to the CNP, which is accounted for in our tight-binding calculation.

Performing such a Fourier analysis for several devices (Supplementary Fig. 9 and Supplementary Note 5) yields much closer agreement with the geometric width *W* (Fig. 3f and horizontal axis of Fig. 1f) than an estimate based only on the zeroth-order Fourier component *c*_0_*W* (first term in equation (2); see vertical axis of Fig. 1f). Fourier spectroscopy of conductance modulations thus allows to disentangle reduced transmission due to scattering at the edges (*c*_0_*W*) from the effective width of the constriction, and proves the relation between the observed Fourier periodicity and the device geometry.

**Figure 4:**
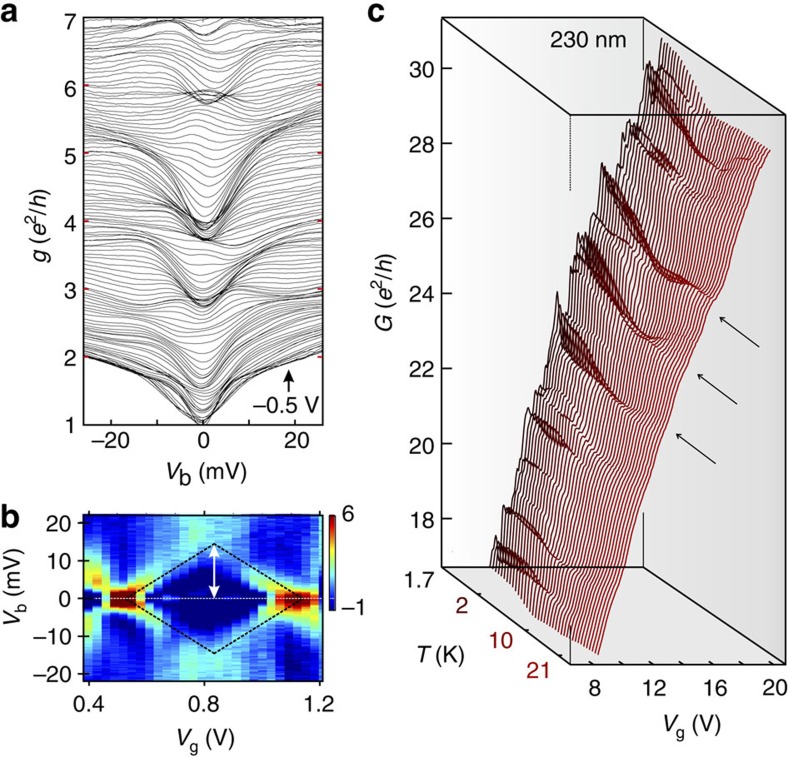
Finite bias and temperature dependence of the quantized conductance. (**a**) Zero *B*-field differential conductance *g* as a function of bias voltage *V*_b_, measured at *T*=6 K, taken at fixed values of back-gate voltage *V*_g_ from −0.5 to 3.0 V in steps of 30 mV (see lower right label). The dense regions correspond to plateaus in conductance. (**b**) Transconductance ∂*g*/∂*V*_g_ in units of *e*^2^/*h*V (see colour scale) as a function of bias and back-gate voltage for a different cool-down of the same device (Supplementary Note 6). At *V*_b_=0, the transitions between conductance plateaus appear as red spots. At finite bias voltage, we observe a diamond-like shape, which provides an energy scale for the sub-band energy spacing Δ*E*≈13.5±2 meV (see dashed black lines and white arrow), which is also in good agreement with the energy scale observed in **a** (Supplementary Note 6). (**c**) Conductance traces as a function of temperature and back-gate voltage. We observe features with different temperature dependencies. Above around 10 K only kinks related to quantized conductance survive (see arrows).

Bias voltage spectroscopy measurements yield an estimate for the energy scale of the size quantization steps^11^,^36^. For example, by analysing finite bias measurements from our smallest constriction device we extract a sub-band energy spacing of Δ*E*=13.5±2 meV near the CNP (Fig. 4a,b, Supplementary Figs 10–12 and Supplementary Note 6). With the geometric width of 230 nm also confirmed by the Fourier spectroscopy (Fig. 3c) we can estimate the Fermi velocity near the CNP as *v*_F_=2*W*Δ*E*/*h*=(1.5±0.2) × 10^6^ m s^−1^. This is a clear signature of a substantially renormalized Fermi velocity in nanostructured graphene, possibly enhanced by electron–electron interaction^37^. Moreover, the extracted energy scales are consistent with the weak temperature dependence of the quantized conductance (Fig. 4c, Supplementary Figs 13 and 14 and Supplementary Note 7).

### Transition from quantized conductance to quantum Hall

**Figure 5:**
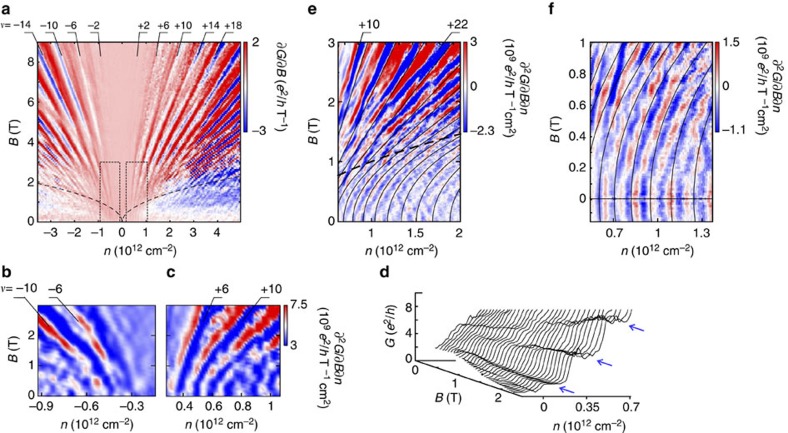
Magnetic-field dependence of the size quantization. (**a**) Landau level fan of the graphene quantum point contact of width *W*≈230 nm, measured at *T*=1.7 K. Landau levels emerge at high magnetic fields. The magnetic-field quantization of Landau level *m* dominates over size quantization as soon as 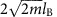 (where the magnetic length 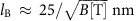) is smaller than the constriction width (*B*-field values above dashed black line). (**b**,**c**) Double-derivative plots of the regions delimited by thin dashed lines in **a** showing the evolution of the lowest quantization plateaus with magnetic field: we observe the full transition from quantized sub-bands (*B*=0 T) to Landau levels at large *B*-field. (**d**) The same magnetic-field evolution is visible in the conductance as a function of magnetic field and charge carrier density for a different cool-down of the same device, also measured at 1.7 K. The blue arrows highlight the expected quantum Hall conductance plateaus at 2, 6 and 10 *e*^2^/*h*. (**e**) Double-derivative plot of the conductance as a function of magnetic field and charge carrier density measured at *T*=6 K. The solid black lines denote the theoretical expectations for the evolution of the size quantization with magnetic field. The thick dashed black line corresponds to the boundary of the Landau level regime, also appearing in **a**. (**f**) Zoom-in of **e** for small magnetic fields *B*≤1 T.

Additional clear fingerprints of size quantization appear in the parametric evolution of the conductance steps^38^ with magnetic field *B*. The transition from size quantization at zero *B*-field to Landau quantization at high magnetic fields occurs when the cyclotron radius *l*_C_ becomes smaller than half the constriction width *W*. For the Landau level *m* the transition should occur at 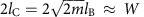 with *l*_B_ the magnetic length. This transition line in the *B*−*n* plane (see black dashed curve in Fig. 5a) agrees well with the onset of Landau level formation in our data (see Supplementary Fig. 15 and Supplementary Note 8 for similar data from a 280-nm constriction device). The evolution of the lowest quantized steps (at *B*=0 T) to the corresponding lowest Landau levels at low temperatures (*T*=1.7 K) can be easily tracked (Fig. 5b,c). At higher temperatures (*T*=6 K) the evolution of quantized sub-bands to Landau levels is observed even for higher conductance plateaus (Fig. 5d,e). For a comparison, we calculate the evolution of size quantization of an infinitely long ribbon of width *W* as function of magnetic field. We take *W*≈230 nm from the SEM data, which leaves no adjustable parameters. Our model (black lines in Fig. 5e,f) reproduces the evolution from the kinks at small fields (*l*_B_≫*W*) to the Landau levels for large fields (*l*_B_<*W*) remarkably well, further supporting the notion that they are, indeed, a signature of size quantization.

## Discussion

We have shown ballistic conductance of confined Dirac fermions in high-mobility graphene nanoconstrictions sandwiched by hBN. Away from the Dirac point, we observe a linear increase in conductance as function of Fermi wave vector with a slope proportional to constriction width. Close to the Dirac point, the charging of localized edge states distorts this linear relation. Superimposed on the linear conductance, we observe reproducible, evenly spaced modulations (kinks). Tight-binding simulations for the device reproduce these structures related to size quantization at the constriction. We can unambiguously identify these ‘kinks' as size quantization signatures by both Fourier spectroscopy at zero magnetic field and their evolution with magnetic field, finding good agreement between theory and experiment.

## Methods

### Experimental methods and details

The hBN–graphene–hBN sandwich structures^8^ have been etched by reactive ion etching in an SF_6_ atmosphere, prior deposition of a ∼10-nm-thick Cr etching mask. Residues of Cr oxide are removed by immersing the samples in a tetramethylammonium hydroxide solution for about 30–35 s. All transport measurements are performed in a four-probe configuration using standard lock-in techniques. Since the distances between the contacted current-carrying electrodes and the voltage probes are small compared with the other length scales of the system, we have an effective two-probe configuration. Importantly, this way we exclude the one-dimensional contact resistances.

### Electrostatic simulations and transport calculations

We simulate the experimental device geometry using a third-nearest neighbour tight-binding ansatz. We rescale our device by a factor of four compared with experiment, to arrive at a numerically feasible geometry. We determine the Green's function using the modular recursive Green's function method^30^,^39^. The local density of states and transport properties can then be extracted by suitable projections on the Green's function. For more technical details see Supplementary Note 9.

## Additional information

**How to cite this article:** Terrés, B. *et al*. Size quantization of Dirac fermions in graphene constrictions. *Nat. Commun.* 7:11528 doi: 10.1038/ncomms11528 (2016).

## Supplementary Material

Supplementary Information Supplementary Figures 1-15, Supplementary Table 1, Supplementary Notes 1-9 and Supplementary References
