# Supplementary material for: Size quantization of Dirac fermions in graphene constrictions
Source: Nat Commun. 2016 May 20;7:11528. doi: 10.1038/ncomms11528 (PMC4876454; doi:10.1038/ncomms11528)
Supplement: Supplementary Information — Supplementary Figures 1-15, Supplementary Table 1, Supplementary Notes 1-9 and Supplementary References [file ncomms11528-s1.pdf]

I. SUPPLEMENTARY FIGURES

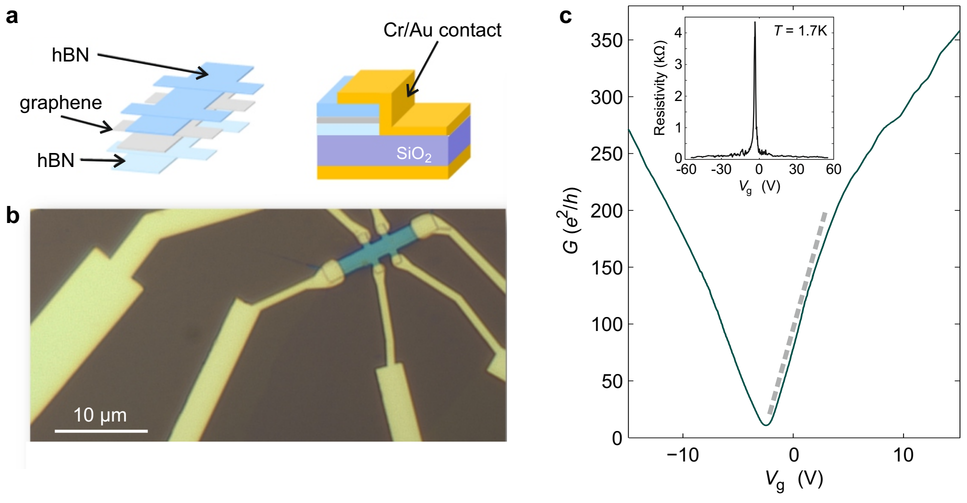

Supplementary Figure 1. Reference hBN-graphene-hBN sandwich Hall bar device. (a) Schematic illustration of the hBN-graphene-hBN sandwich structure and nature of the quasi-one-dimensional graphene-metal (Cr/Au) contact. **(b)** Optical image of an etched and contacted ∼1 μm-wide hBN-graphene-hBN sandwich Hall bar device. **(c)** Four-terminal conductance as a function of back gate voltage V_g measured at a constant current of 50 nA and a temperature of 16 K. From the linear slope near the charge neutrality point (see dashed line) we extract a carrier mobility of around 150.000 $cm^2\ V^{-1}s^{-1}$. The inset shows the four-terminal resistivity as a function of gate voltage at lower temperature (1.7 K).

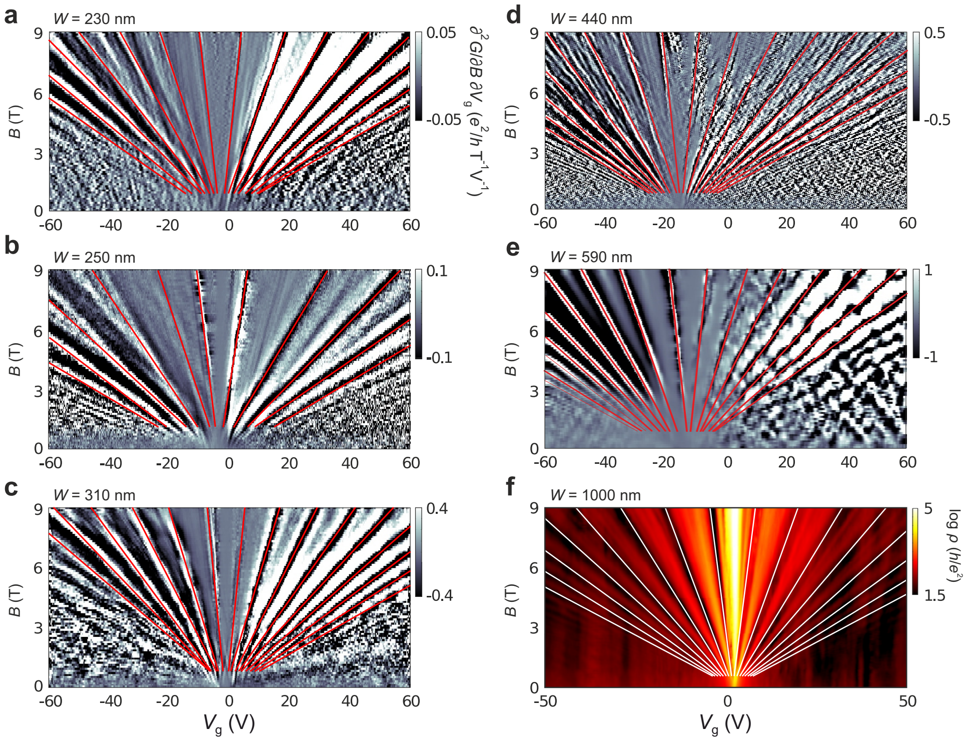

Supplementary Figure 2. Landau fan and capacitive coupling. (a)-(e) Second derivative of the longitudinal conductance $\partial^2 G/\partial V_g \partial B$ as a function of magnetic field B and back-gate voltage V_g for six different devices with different widths. The red lines follow the evolution of the Landau levels. The slopes of the lines are proportional to the capacitive coupling α. **(f)** The longitudinal resistivity ρ as a function of B and V_g provide an alternative way to extract α from the position of the Landau levels, marked by white lines.

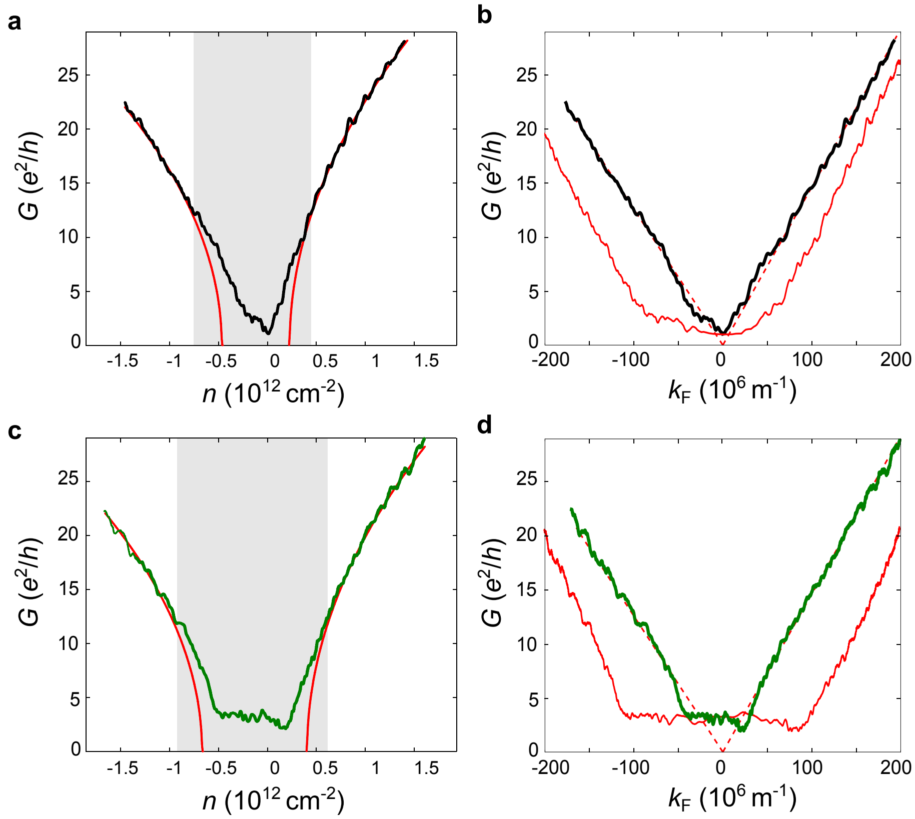

Supplementary Figure 3. Deviation from the ideal ballistic conductance for the 230 nm-wide graphene constrictions. (a) Low-bias four-terminal conductance G as a function of charge carrier density n. The red solid lines are fits to a simple capacitive coupling model (Eq. 2) valid at high carrier densities for the holes and electrons regime, respectively. Deviations appear in the gray-shaded region around the charge neutrality point. **(b)** Conductance G of panel (a) as a function of k_F using the linear density of states of ideal graphene (red solid line), or including a finite density of trap states (Eq. 4) around the Dirac point (black solid line). The linear relation between $G(k_\mathrm{F})$ and k_F expected for ideal graphene is shown as a dashed red line. **(c)** and **(d)** Corresponding to (a) and (b) but for a different cool-down of the same constriction. After exposing the sample to ambient conditions, the number of charge traps responsible for the flat area around the Dirac point increased significantly.

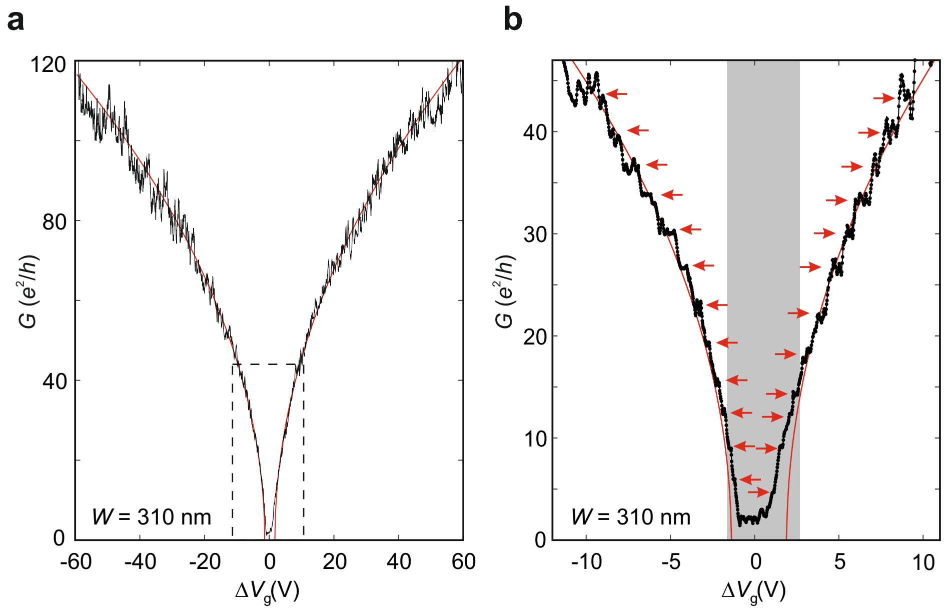

Supplementary Figure 4. Kinks in the back-gate characteristics of the 310 nm-wide graphene constriction (a) Low-bias four-terminal conductance G as a function of back gate voltage V_{g}, measured at $T = 2$ K. The ideal Landau-Büttiker model of conductance $G \propto \sqrt{n}$ is marked in red. **(b)** Close-up of the conductance G inside the dashed-line region of panel a. The reproducible kinks are clearly visible (marked by red arrows). The shaded gray region denote deviations from the ideal Landauer model (red trace).

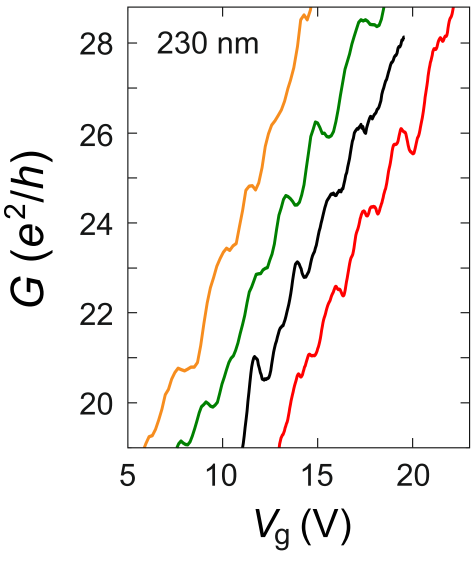

Supplementary Figure 5. Cool-down dependence of the kinks for the 230 nm-wide graphene constriction I. Four-terminal conductance G as a function of back gate voltage V_{g} for four different cool-downs of the 230 nm-wide graphene constriction. The traces are shifted horizontally for clarity.

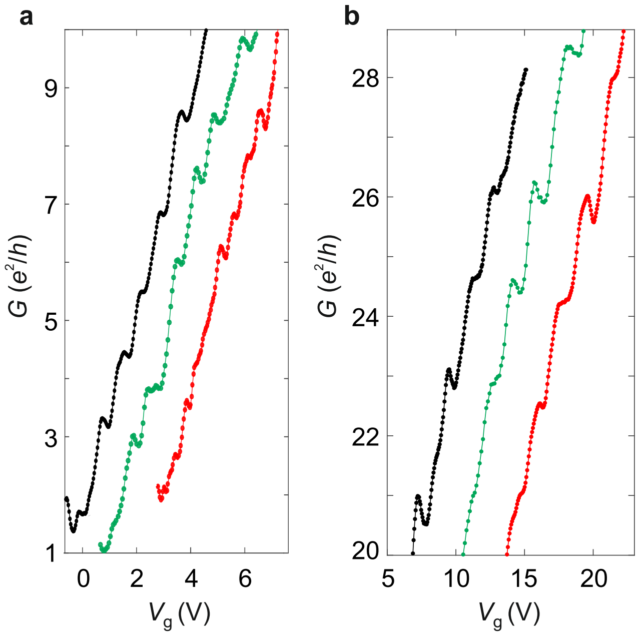

Supplementary Figure 6. Cool-down dependence of the kinks for the 230 nm-wide graphene constriction II. (a) and **(b)** Four-terminal conductance G as a function of back gate voltage V_g for different cool-downs of the 230 nm-wide graphene constriction at low and high charge carrier densities (panels a and b, respectively). The traces are shifted horizontally for clarity.

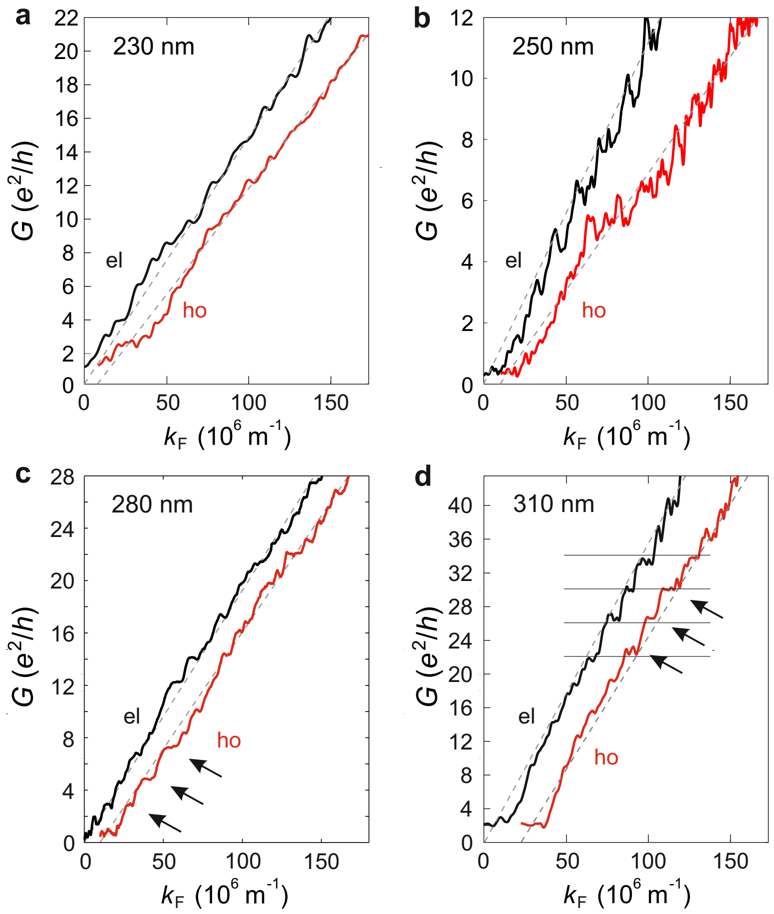

Supplementary Figure 7. Width dependence of the kinks in conductance. Four-terminal conductance G as a function of back gate voltage V_g for four different devices of widths 230 nm (**a**), 250 nm (**b**), 280 nm (**c**) and 310 nm (**d**). The transmission traces are shown in black (red) for electrons (holes) as a function of rescaled k_F (see main text). The arrows point to kinks where the conductance jumps by about $c_0 \times \frac{4e^2}{h}$, with c_0 as measure for the overall transmission of the device, see Supplementary Equation 3; $c_0 \approx 0.95$ for the 310 nm constriction. The traces are shifted horizontally for clarity.

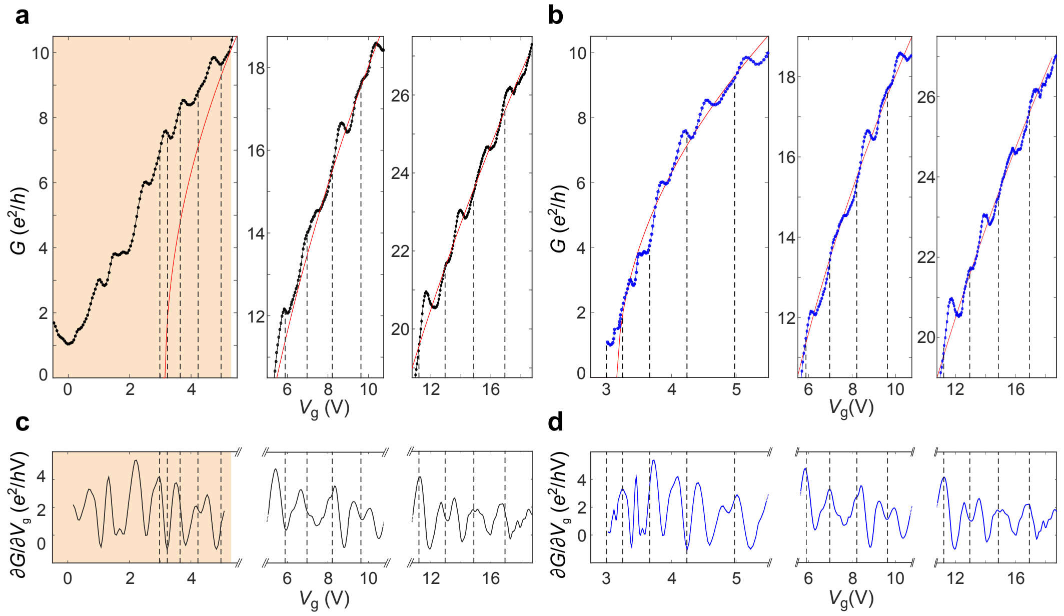

Supplementary Figure 8. Back-gate characteristics of the energy subbands of the 230 nm-wide graphene constriction. **(a)** Low-bias four-terminal conductance G as a function of back-gate voltage V_g. The theoretical position of the subbands in the V_g-axis is indicated by vertical dashed lines. Close to the Dirac point (leftmost subpanel) measurements deviate from the ideal Landau model $G \propto \sqrt{V_\mathrm{g}}$ shown in red (orange-shaded region). **(b)** Same as (a) after rescaling of the charge carrier density (Equation 3). The vertical dashed lines indicating the theoretical position of the subbands matches now the positions of kinks. **(c)** Derivative plot $\partial G/\partial V_\mathrm{g}$ of the conductance trace shown in panel (a). The correlation between the expected position of the subbands (vertical dashed lines) and measurements holds only at high carrier densities. **(d)** Derivative plot $\partial G/\partial V_\mathrm{g}$ of the conductance trace in panel (b).

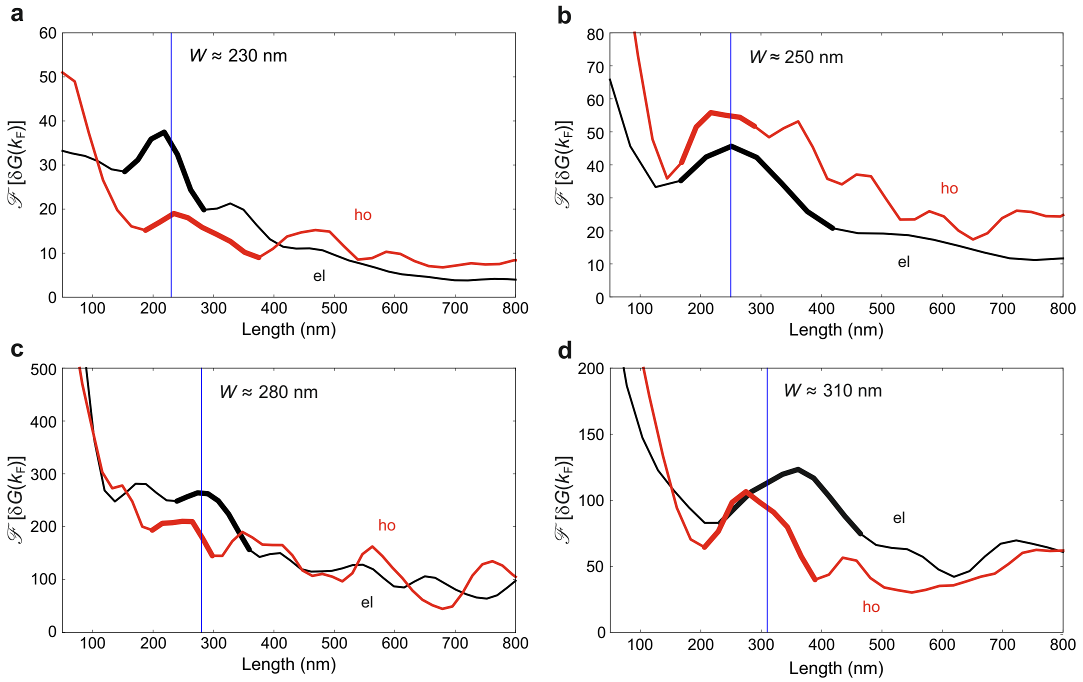

Supplementary Figure 9. Fourier transform of the conductance. Fourier transform of the electron (black) and hole (red) conductance for the devices of width 230 nm (**a**), 250 nm (**b**), 280 nm (**c**) and 310 nm (**d**). The widths extracted from the Fourier analysis $W_{\rm FT}$ (peaks in $\mathscr{F}[\delta G(k_{\rm F})]$) are in good agreement with the widths extracted from SEM images (blue vertical lines). The extracted widths $W_{\rm FT}$ and associated errors bars are shown in Figure 3f.

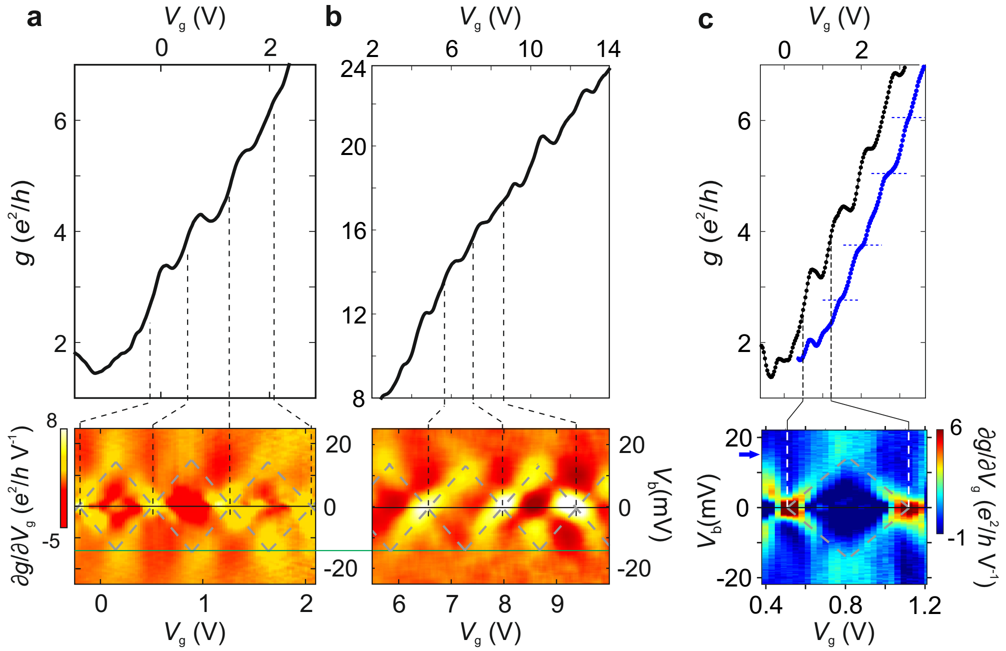

Supplementary Figure 10. Bias spectroscopy of the 230 nm-wide graphene constriction. **(a)** Differential conductance g (upper panel) and differential transconductance $\partial g/\partial V_\mathrm{g}$ (lower panel) as a function of back gate V_g and bias V_b voltages, measured at $B = 0$ T and $T = 6$ K. The differential conductance g (top panel) is measured at $V_\mathrm{b} = 0$ V in the low carrier density range. The vertical black dashed lines indicate the position of the analyzed subbands. The transconductance $\partial g/\partial V_\mathrm{g}$ (bottom color-scaled panel), of the data shown in the upper panel, is measured as a function of an applied bias voltage V_b. The kinks are characterized by high values (yellow color) of transconductance. The diamond structures are highlighted by dashed gray diamonds. We extract an average subband spacing $\Delta E \approx 13.5 \pm 2$ meV (green line). **(b)** Same as panel **(a)** measured at high carrier densities. **(c)** Same as panel **(a)** for a second cool-down of the same device. The blue trace represents the differential conductance g measured at $V_\mathrm{b} = 15\,\mathrm{mV}$ (see blue arrow in lower colored panel). The horizontal dashed blue lines highlight the levels of conductance of the intermediate kinks, visible (blue conductance trace) for energies above the subband spacing, e.g. $E \approx 15\,\mathrm{meV} > \Delta E$ (blue arrow in lower colored panel).

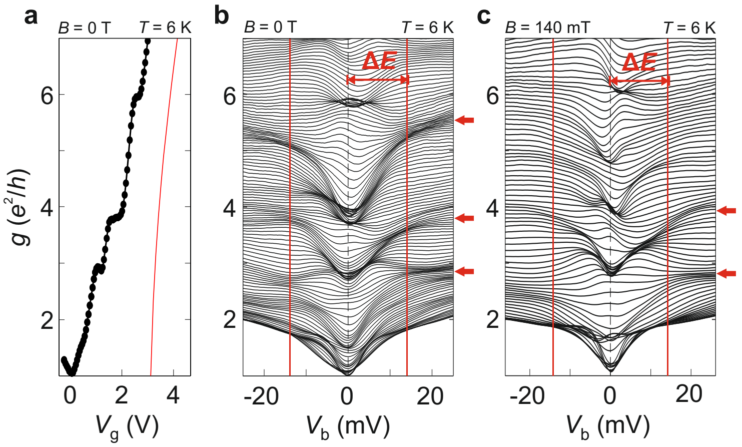

Supplementary Figure 11. Finite bias spectroscopy of the 230 nm-wide graphene constriction. **(a)** Differential conductance g as a function of back-gate voltage V_g, measured at $V_b = 0$ V, $B = 0$ T and $T = 6$ K. The red solid line shows the ballistic model of conductance, fitted at high carrier densities. **(b)** Differential conductance g as a function of source-drain voltage V_b. The traces are taken at fixed values of back-gate voltage V_g from -0.5 V (lower trace) to 3.0 V (upper trace) in steps of 30 mV. The dense regions correspond to kinks in conductance. The intermediate kinks at high bias voltage are marked by red arrows. The subband spacing $\Delta E \approx 13.5 \pm 3$ meV is highlighted by a vertical red line. **(c)** Differential conductance g as a function of source-drain voltage V_b measured at $B = 140$ mT. The intermediate kinks at high bias voltage are marked by red arrows. We extract an equal subband spacing as in panel b, $\Delta E \approx 13.5 \pm 3$ meV (vertical red line).

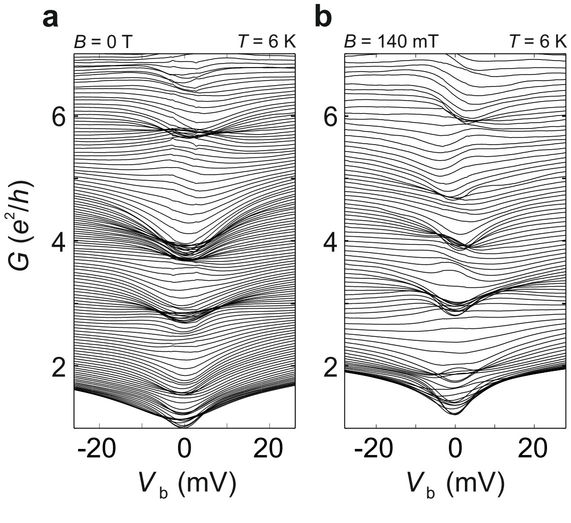

Supplementary Figure 12. Finite DC bias spectroscopy of the 230 nm-wide graphene constriction. **(a)** DC spectroscopy of the same device as in Supplementary Figure 11. **(b)** Conductance G as a function of DC source-drain voltage V_b measured at $B = 140$ mT and $T = 6$ K, for the same device as in panel a.

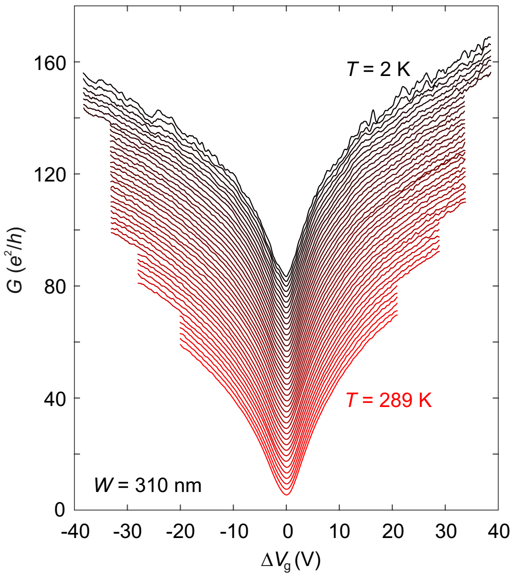

Supplementary Figure 13. Temperature dependence of the back-gate characteristics for the 310 nm-wide graphene constriction. Low-bias back-gate dependent four-terminal conductance G as a function of temperature T. The traces are shifted in the conductance axis for clarity. Temperature is recorded from $T = 2$ K (black trace) up to room-temperature ($T = 289$ K, red trace), in steps of 7 K.

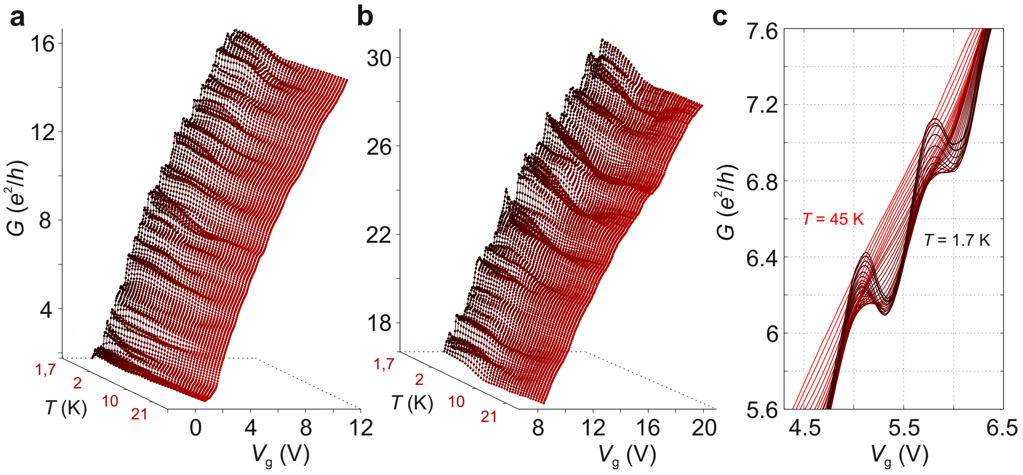

Supplementary Figure 14. Temperature dependence of the conductance kinks for the 230 nm-wide constriction. (a) and **(b)** Four-terminal conductance G as a function of back gate voltage V_g and temperature T, at low (panel a) and high (panel b) carrier densities. Measurements are recorded at temperatures from $T = 2$ K to $T = 24$ K in steps of 0.7 K. **(c)** Zoom-in of the temperature evolution of the shape of the kinks.

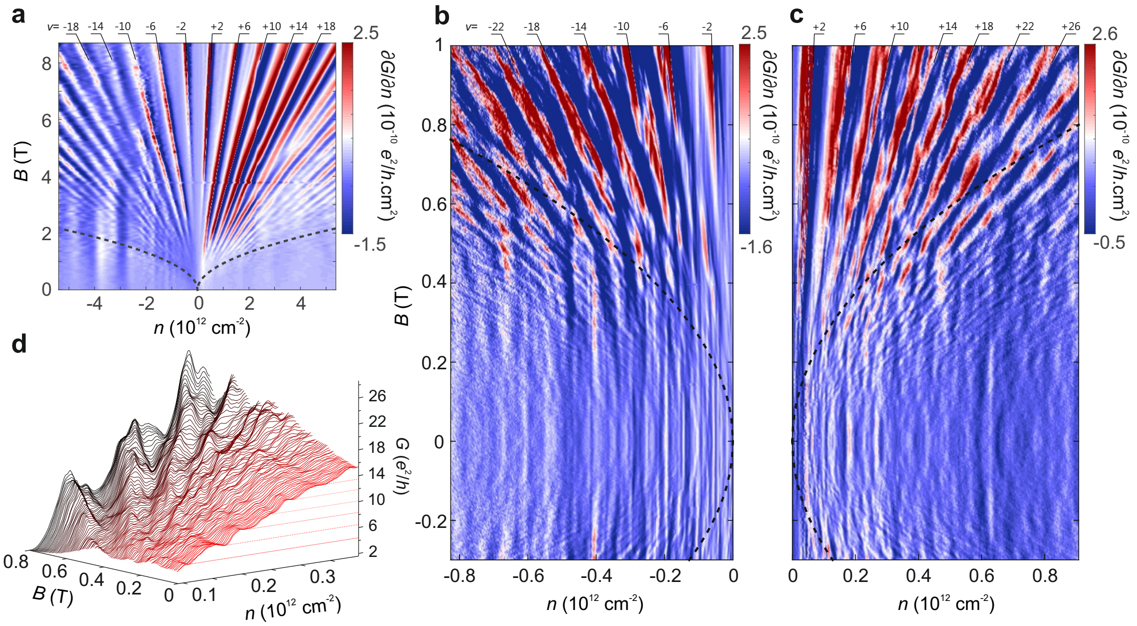

Supplementary Figure 15. Magnetic-field dependence of the size quantization for the 280 nm-wide graphene constriction. **(a)** Landau level fan of the 280 nm-wide graphene constriction. **(b)** and **(c)** High resolution double derivative plots, measured at low magnetic fields $B \leq 1$ T, in the low-carrier density range for the hole- and electron-regimes, respectively. In panels a, b and c the black dashed line denotes the boundary above which the magnetic field quantization of Landau level m dominates over size quantization, i.e. when $2\sqrt{2m}l_{\mathrm{B}} < W$. **(d)** Evolution of the conductance traces as a function of charge carrier density n and magnetic field B. The B-field step size between traces is 8 mT. The data was measured at $T = 1.7$ K.

II. SUPPLEMENTARY TABLES

SEM width W (nm)	α (10^{10} cm^{-2}V^{-1})
1000	7.00
850	5.80
590	6.75
440	6.90
310	7.00
280	7.20
250	5.40
230	7.15

Supplementary Table 1. Lever arm values α for eight different devices extracted from the Landau level fan measurements (see Supplementary Figure 2).

III. SUPPLEMENTARY NOTES

Supplementary Note 1. Sample quality

The field-effect carrier mobility in our sandwich devices is on the order of 150.000 $\mathrm{cm}^2\ \mathrm{V}^{-1}\mathrm{s}^{-1}$. This high sample quality is thanks to advances in sample fabrication, in particular the van-der-Waals stacking process: the graphene is fully encapsulated in hBN, resulting in significantly improved sample quality. We extract the mobility from a one μm-wide Hall bar device fabricated in the very same batch as our graphene constrictions (see Supplementary Figure 1). The dark blue trace in Figure 1d of the main manuscript is taken from this Hall bar device. As all traces from the constrictions with different widths (some of them carved out from the same hBN-graphene-hBN sandwich) lie systematically below the Hall bar trace, we exclude bulk scattering as limiting process in our devices. Independently, we have shown recently in a collaboration with A. Morpurgo, F. Guinea and coworkers[1] that in our high-quality devices the carrier mobility is not limited by charge impurity and short-range scattering but rather by nanometer-scale strain variations giving rise to long-range scattering with allowed pseudospin flips. We expect that the same limitations on the mean free path also apply to our graphene constriction devices.

Supplementary Note 2. Extraction of the gate lever arm α

Measurements of Landau levels in graphene as a function of back gate voltage V_g and magnetic field B (see Supplementary Figure 2) allow for an independent determination of the gate coupling (or lever arm) α. The Landau level spectrum for massless Dirac fermions in graphene is given by

$$E_m(B) = \mathrm{sgn}(m) v_\mathrm{F}\sqrt{2|e|\hbar|m|B}, \quad m \in \mathbb{Z}_0, \tag{1}$$

where v_F is the Fermi velocity and m is the quantum number of the corresponding Landau level. Assuming a perfect linear dispersion and a constant capacitive gate coupling leads to the following relation between energy E and back gate voltage

$$E = \hbar v_\mathrm{F} k_\mathrm{F} = \hbar v_\mathrm{F}\sqrt{\pi\alpha\Delta V_\mathrm{g}}, \tag{2}$$

where $\Delta V_\mathrm{g} = V_\mathrm{g} - V_\mathrm{g}^0$, and V_g^0 is the gate voltage at the charge neutrality point. As a result, the Landau levels in the B - V_g plane form straight lines, i.e. $B_m = C_m \Delta V_\mathrm{g}$, where the slope $C_m = \alpha h/4me$ is Landau level index (m) dependent and proportional to the capacitive coupling α (see red lines in Supplementary Figure 2a-e).

The onset of each Landau level can be resolved by taking the mixed second derivative of the longitudinal conductance G with respect to V_g and B, i.e. $\partial^2 G/\partial V_\mathrm{g}\partial B$. The positions of the Landau levels coincide with the minima/maxima of the derivative on the electron/hole side (see Supplementary Figure 2a-e, where the local minima/maxima coincide with red lines). Alternatively, the

Landau levels can be determined from the minima of the longitudinal resistivity ρ (marked in white in Supplementary Figure 2f). Note that C_m is independent of the Fermi velocity, experimental determination of which is rather difficult. Supplementary Table 1 summarizes the extracted values of α for the different devices.

Supplementary Note 3. Linearization of G as a function of k_F

For a known gate coupling α, one can evaluate the measured conductance $G(V_\mathrm{g})$ as a function of k_F, using the standard constant capacitive coupling model $k_\mathrm{F} = \sqrt{\pi\alpha\Delta V_\mathrm{g}}$. Following the Landauer theory of conductance through a constriction of finite width W, the averaged conductance $G^{(0)}(V_\mathrm{g})$ features a square-root dependence on V_g,

$$G^{(0)} = \frac{4e^2}{h}\left(\frac{c_0 W k_\mathrm{F}}{\pi} - \frac{1}{2}\right) = \frac{4e^2}{h}\frac{c_0 W}{\pi}\sqrt{\pi\alpha(V_\mathrm{g} - V_\mathrm{g}^0)} - \frac{2e^2}{h}. \tag{3}$$

A closer look at the traces from two different cool-downs of the narrowest device with $W =$ 230 nm (Supplementary Figures 3a and 3c) reveals a systematical deviation from the expected square-root dependence of G (Supplementary Equation 3) at low carrier concentrations, i.e for $n < 0.45\times10^{12}$ cm^{-2} on the electron side and $n < 0.75\times10^{12}$ cm^{-2} on the hole side (Supplementary Figure 3a). This deviation becomes more pronounced closer to the charge neutrality point (see shaded area in Supplementary Figures 3a and 3c). In the ballistic region, i.e., far from the charge neutrality point, we can use Supplementary Equation 3, with α extracted from the Landau level fan, and fit parameters $V_\mathrm{g}^{0,\mathrm{e}}$ for the electron (e) and $V_\mathrm{g}^{0,\mathrm{h}}$ for the hole (h) side. As expected, the conductance G evolves linearly as function of k_F in the ballistic regime (see red traces in Supplementary Figures 3b and 3d), but large deviations between data and model become apparent close to the charge neutrality point. We conclude that a linear model using a constant gate coupling is not directly applicable to our graphene constriction devices. Instead, one needs to account for the additional charge carrier trap states n_T (see main text), modifying the relation between back-gate voltage and Fermi wave number according to

$$\alpha(V_\mathrm{g} - V_\mathrm{g}^0) = \alpha\Delta V_\mathrm{g} = k_\mathrm{F}^2\pi^{-1} + n_\mathrm{T}\left(\Delta V_\mathrm{g}\right). \tag{4}$$

Using Supplementary Equation 4, we obtain an implicit mapping $k_\mathrm{F}(\Delta V_\mathrm{g})$, which depends on the functional form of $n_\mathrm{T}(\Delta V_\mathrm{g})$ and accounts for the modified density of states in the constriction,

$$k_\mathrm{F}(\Delta V_\mathrm{g}) = \sqrt{\pi\alpha\Delta V_\mathrm{g} - \pi n_\mathrm{T}\left(\Delta V_\mathrm{g}\right)}. \tag{5}$$

We conjecture that the strong cool-down dependence seen in the different traces of Supplementary Figure 3 are due to modifications in the trap state densities as the sample was exposed to air[2]. As the graphene layer in our hBN-graphene-hBN sandwich can only interact with air at the edges, edge states presumably strongly contribute to n_T. Indeed, tight-binding simulations of the constriction geometry (see main text, Figure 2c,e) yield a clustering of localized edge states close to the Dirac point. Accounting for n_T by Supplementary Equation (5) should recover the

linear relation between Fermi wave number and conductance. We can thus determine n_T from the measured conductance: we assume a Gaussian distribution of the density of trap states, and fit the width, position and height of the Gaussian distribution by minimizing deviations of the rescaled conductance $G[(k_\mathrm{F}(\Delta V_\mathrm{g})]$ from the linear conductance $G^{(0)}(k_\mathrm{F})$ of Supplementary Equation (3), see green/black traces in Supplementary Figures 3b and 3d. Note that this procedure assumes that any other sources for a deviation from a linear relation between k_F and G (due to, e.g., many-body effects) are small compared to the contribution from trap states n_T.

Supplementary Note 4. Reproducibility of kink signatures

We find regular kink structures in the conductance trace of our constriction devices (see, e.g., arrows in Supplementary Figure 4b). These kinks are well reproducible for different cool-downs of the same device (see Supplementary Figures 5, 6), and appear in conductance data of several different devices (see Supplementary Figure 7).

Analyzing the position of kinks as a function of back-gate voltage offers an independent check of the trap state density n_T. In a first order approximation, the band structure of a graphene constriction of width W can be described as a collection of one-dimensional subbands originating from the quantization of the wave vector perpendicular to the transport direction,

$$k_\perp = \pm \left| M + \beta \right| \pi / W, \tag{6}$$

where $M = 0, \pm 1, \pm 2, \ldots$ is an integer associated with the subband index (both signs emerge due to the presence of two cones), and $0 \leq |\beta| < 0.5$ is a Maslov index related to the boundary conditions at the edges (for simplicity we use $\beta = 0$, i.e. a zigzag ribbon). Within the energy range where the ballistic model (see red trace in Supplementary Figure 8) fits the conductance trace, the theoretical position of the subbands (marked by vertical black dashed lines in Supplementary Figure 8) for a 230 nm-wide graphene constriction ($V_\mathrm{g}^M = \pi M^2/\alpha W^2$, $M = 1, 2, \ldots$) are in good agreement with the kinks in the conductance (see Supplementary Figure 8a). The agreement between model and data is also visible in the derivative of the conductance $\partial G/\partial V_\mathrm{g}$ (see Supplementary Figure 8b). Close to the charge neutrality point though, the kink signatures do not appear to follow the theoretical position of the subbands (vertical black dashed lines in Supplementary Figures 8a, and 8b). Upon rescaling k_F according to Supplementary Equation (5) (independently determined from the average transmission), the kinks are shifted, in good agreement with the quantization model (see comparison between dashed vertical lines and the position of the kinks in Supplementary Figures 8c and 8d). In summary, we find that the rescaling according to Supplementary Equation (5) will (i) realign similar, reproducible kink-structures of different cool-downs on the k_F axis and (ii) shifts the kink positions to fit the simple quantization model of Supplementary Equation (6).

Supplementary Note 5. Fourier spectroscopy of transmission data

Once the conductance is represented as a function of k_F, the Fourier transform of $\delta G(k_\mathrm{F})$ offers alternative information on the quantized conductance through the constriction. If the regular kinks we identify in our conductance data, indeed, correspond to size quantization signatures, we can extract the constriction width from the first peak of the Fourier transform. Comparison between the first peak in the Fourier transform of the measured conductance $G(k_\mathrm{F}) - G^{(0)}(k_\mathrm{F})$ of four constriction devices (see Supplementary Figure 9 and Figure 4 in the main text) to the geometric width W of the constriction, yields good agreement (see also Figure 3f of the main text).

Supplementary Note 6. Bias spectroscopy

Using bias spectroscopy we can extract the energy scale associated with the regular kink pattern. The differential conductance $g = dI/dV$ (Figure 4, Supplementary Figures 10 and 11) is measured from an AC excitation voltage $V_\mathrm{AC} = 250\ \mu\mathrm{V_{PP}}$, using standard Lock-In techniques. We analyze six diamonds associated with kinks at the low- and high-conductance ranges (see Supplementary Figure 10). Extraction of the energy scale from the derivative of the differential conductance (color panels) yields $\Delta E = 13.5 \pm 2$ meV leading to $v_\mathrm{F} = (1.5 \pm 0.2) \times 10^6$ m s^{-1}. Variations in the data are due to temperature effects, potential variations and uncertainties in determining the exact extensions of the diamonds. All six extracted diamonds are taken from energy regions where size quantization signatures are clearly visible and reproducible - we are thus confident that the sample is in the quantum point contact regime for all six diamonds. Note that modifications of the gate-lever arm do not affect the bias spectroscopy data since all energy scales are extracted from the bias voltage axis (V_b), which represents a direct energy-scale.

We extract similar values of subband spacing ($\Delta E \approx 13.5 \pm 2$ and 13.5 ± 3 meV) in a second (Figure 4b of the main text and Supplementary Figure 10c) and a third (Figure 4a of the main text and Supplementary Figure 11) cool-down of the same device. The value of subband spacing is additionally confirmed at finite magnetic field (Supplementary Figure 11c). We note that, at $B = 140$ mT, the quantized subbands are still caused by geometric confinement rather than magnetic confinement (i.e., due to the quantum Hall effect).

Moreover, half-conductance kinks[3,4] are expected to emerge for a bias window $e\,V_\mathrm{b}$ greater than the subband spacing. Indeed, additional kinks at intermediate values of conductance are observed (horizontal dashed blue lines in Supplementary Figure 10c and red arrows in Supplementary Figure 11b,c). The observation of these intermediate kinks confirms the confinement nature of the observed kinks in conductance[3,5,6].

To check against any spurious contribution from the AC measurement technique, the bias spectroscopy measurements have been repeated in a DC configuration (Supplementary Figure 12). The conductance $G = I/V_\mathrm{b}$ is obtained from a symmetrically applied source-drain DC bias voltage V_b. Although the resolution of the DC conductance G (Supplementary Figure 12) is not sufficient

to extract the subband spacing ΔE, the conductance kinks are still visible at identical values of conductance as in the AC measurements (Supplementary Figure 11).

Supplementary Note 7. Temperature dependence

In Supplementary Figures 13 and 14 we show additional data on the temperature dependence of our transport data highlighting both (i) the high quality of our samples and (ii) the energy scale and stability of the observed kink features.

Supplementary Note 8. Evolution of size quantization with magnetic field

We provide an additional data set for the magnetic-field evolution of the size quantization signatures from the 280 nm-wide graphene constriction in Supplementary Figure 15. We find the same transition from size-quantization signatures, at low magnetic fields, to the Landau level regime, at high magnetic fields, as in the sample discussed in the main text (see Figure 5 of main manuscript).

Supplementary Note 9. Theoretical treatment

We use a third nearest neighbor tight-binding approach to simulate the constriction. We pattern the device edge using the experimental geometry determined from SEM, and a correlated random fluctuation to simulate microscopic roughness. We rescale our device by a factor of four compared to the experiment, to arrive at a numerically feasible system size. Such a rescaling by a factor of four ensures that all relevant length scales of the problem (e.g., device geometry, Fermi wavelength, magnetic length and correlation length of the edge roughness) are still much larger than the discretization length of the numerical graphene lattice, allowing to extrapolate simulation data to the experimental result[7]. We use a correlation length of 5 nm and an average disorder amplitude of 13 nm. We determine the Green's function, $\mathcal{G}(r, r')$, of the device using the modular recursive Green's function method[8,9]. The local density of states, $\rho(r, E)$, is given by $\rho(r, E) \propto \mathrm{Im}[\mathcal{G}(r, r; E)]$. Calculations were performed on the Vienna Scientific Cluster 3. To determine the transport properties of the device, we attach two leads of width D on each side of the experimental contact regions, and calculate the total transmission. To avoid residual effects due to the fixed lead width used in the computation, we average over five different randomly chosen lead widths $D \in [60, 80]$ nm.

To determine the evolution of subbands in a constriction of width W with magnetic field, we calculate the band structure of a perfect zigzag graphene nanoribbon of width W as a function of

magnetic field. We include the magnetic field via a Peierls phase factor. The subband positions are extracted from the minima of each band in the bandstructure of the ribbon.

SUPPLEMENTARY REFERENCES

[1] Couto, N.J., Costanzo, D., Engels, S., Ki, D.K., Watanabe, K., Taniguchi, T., Stampfer, C., Guinea, F. & Morpurgo, A.F., Random strain fluctuations as dominant disorder source for high-quality on-substrate graphene devices. *Phys. Rev. X* **4**, 041019 (2014).

[2] Woessner, A. et al., Near-field photocurrent nanoscopy on bare and encapsulated graphene. *Nat. Commun.* **7**, 10783 (2016).

[3] Van Weperen, I., Plissard, S.R., Bakkers, E.P.A.M., Frolov, S.M. & Kouwenhoven, L.P., Quantized conductance in an InSb nanowire. *Nano Lett.* **13**, 387391 (2013)

[4] Kouwenhoven, L.P., Van Wees, B.J., Harmans, C.J.P.M., Williamson, J.G., Van Houten, H., Beenakker, C.W.J., Foxon, C.T. & Harris, J.J., Nonlinear conductance of quantum point contacts. *Phys. Rev. B* **39**, 8040-8043 (1989).

[5] Patel, N.K., Nicholls, J.T., Martn-Moreno, L., Pepper, M., Frost, J.E.F., Ritchie, D.A. & Jones, G.A.C., Properties of a ballistic quasi-one-dimensional constriction in a parallel high magnetic field. *Phys. Rev. B* **44**, 10973 (1991).

[6] Glazman, L.I. & Khaetskii, A.V., Nonlinear quantum conductance of a point contact. *JETP Lett.* **48**, 591-595 (1988).

[7] Liu, M.H., Rickhaus, P., Makk, P., Tovari, E., Maurand, R., Tkatschenko, F., Weiss, M., Schönenberger, C. & Richter, K., Scalable tight-binding model for graphene. *Phys. Rev. Lett.* **114**, 036601 (2015).

[8] Libisch, F., Rotter, S., & Burgdörfer, J., Coherent transport through graphene nanoribbons in the presence of edge disorder. *New Journal of Physics* **14**, 123006 (2012).

[9] Rotter, S., Tang, J.Z., Wirtz, L., Trost, J. & Burgdörfer, J., Modular recursive Greens function method for ballistic quantum transport. *Phys. Rev. B* **62**, 1950-1960 (2000).
